# Chagas disease: national survey of seroprevalence in children under five years of age conducted in 2008

**DOI:** 10.1590/0074-02760160407

**Published:** 2017-03-27

**Authors:** Graciela Russomando, Blanca Cousiño, Zunilda Sanchez, Laura X Franco, Eva M Nara, Lilian Chena, Magaly Martínez, María E Galeano, Lucio Benitez

**Affiliations:** 1 Universidad Nacional de AsunciónUniversidad Nacional de AsunciónInstituto de Investigaciones en Ciencias de la SaludLaboratorio de Biología MolecularAsunciónParaguayUniversidad Nacional de Asunción, Instituto de Investigaciones en Ciencias de la Salud, Laboratorio de Biología Molecular, Asunción, Paraguay; 2 Ministerio de Salud Pública y Bienestar SocialAsunciónParaguayMinisterio de Salud Pública y Bienestar Social, Servicio Nacional de Erradicación del Paludismo, Asunción, Paraguay

**Keywords:** Chagas disease, domestic *Triatoma infestans*, Trypanosoma cruzi, interruption of transmission, seroprevalence

## Abstract

**BACKGROUND:**

Since the early 1990s, programs to control Chagas disease in South America have focused on eradicating domiciliary *Triatoma infestans*, the main vector. Seroprevalence studies of the chagasic infection are included as part of the vector control programs; they are essential to assess the impact of vector control measures and to monitor the prevention of vector transmission.

**OBJECTIVE:**

To assess the interruption of domiciliary vector transmission of Chagas disease by *T. infestans* in Paraguay by evaluating the current state of transmission in rural areas.

**METHODS:**

A survey of seroprevalence of Chagas disease was carried out in a representative sample group of Paraguayans aged one to five years living in rural areas of Paraguay in 2008. Blood samples collected on filter paper from 12,776 children were tested using an enzyme-linked immunosorbent assay. Children whose serology was positive or undetermined (n = 41) were recalled to donate a whole blood sample for retesting. Their homes were inspected for current triatomine infestation. Blood samples from their respective mothers were also collected and tested to check possible transmission of the disease by a congenital route.

**FINDINGS:**

A seroprevalence rate of 0.24% for *Trypanosoma cruzi* infection was detected in children under five years of age among the country’s rural population. Our findings indicate that *T. cruzi* was transmitted to these children vertically. The total number of infected children, aged one to five years living in these departments, was estimated at 1,691 cases with an annual incidence of congenital transmission of 338 cases per year.

**MAIN CONCLUSION:**

We determined the impact of vector control in the transmission of *T. cruzi*, following uninterrupted vector control measures employed since 1999 in contiguous *T. infestans*-endemic areas of Paraguay, and this allowed us to estimate the degree of risk of congenital transmission in the country.

Chagas disease, a zoonosis transmitted by triatomine insect vectors, remains a major public health problem in most Latin American countries. In the 21 countries where the disease is endemic, an estimated 7.7 million persons are currently infected by the protozoan, *Trypanosoma cruzi*. Each year, approximately 41,200 new vector-borne cases of *T. cruzi* infection are reported, and 14,400 infants are born with congenital Chagas disease (OPS 2006). In South America, *Triatoma infestans* is the domestic vector responsible for the spread of the chagasic epidemic (WHO 2002). In recent years, epidemiological surveillance of *T. cruzi* infections has improved markedly in many endemic countries owing to blood bank screening and vector-control programs. Since the early 1990s, Chagas disease control programs in South America have focused on eradicating domiciliary *T. infestans*, the main vector of the pathogenic protozoan (WHO 2002, OPS 2006). These efforts have proven to be successful in Uruguay, Chile, Brazil, and the Oriental Region of Paraguay (comprising 98% of the country’s total population), all of which have been declared vector-transmission-free by the Pan American Health Organization. Following the success of these National Programs directed at domiciliary vector control and surveillance, prevalence rates in younger age groups have been regressing in many areas (Dias et al. 2002, Schofield et al. 2006, Moncayo & Silveira 2009).

The interruption of domiciliary vector transmission is the highest level of control that can be achieved with information obtained through entomological indicators. In Paraguay, the baseline infestation rate and infection in humans was initially determined between 1982 and 1986. It was shown that 60% of the departments in the country (mainly in rural areas) had an infestation rate of *T. infestans* ranging from 11-30%. In 1986, a national serological survey of *T. cruzi* infection reported a seroprevalence rate of 20-22% in rural areas (Rojas de Arias et al. 1984, 1999).

From 1999-2007, Paraguay was in a technologically prominent position for its use of chemical control agents and entomological surveillance (Silveira 2001, Silveira & Sanches 2003, OPS 2004). These methods led to a significant reduction in the number of *T. infestans*-infested dwellings and the incidence/prevalence of Chagas disease in broad endemic areas (Dias et al. 2002, Schofield et al. 2006, Dias 2007). A five-year project financed in 2002 by the Canadian agency, CIDA, was launched to establish horizontal surveillance systems with the participation of the local community. The strategy involved local technical teams from the National Program performing active searches for vectors in infested villages from rural areas and responsibility for the training of local community leaders in the passive surveillance of vectors in households within their community. Additional local participation was achieved with the organisation of a “Chagas School Week”, sponsored together with the Ministry of Education and Culture.

Seroprevalence studies of the chagasic infection are included as part of the vector control programs, and they are essential to characterise the baseline status, assess the impact of vector control measures, and monitor the prevention of vector transmission. These studies, carried out by sampling, do not necessitate any population census unless the area under study has a very small population (fewer than 100 people). To confirm the interruption of domiciliary transmission, seroprevalence studies in children aged less than five years are advised every five to 10 years (Silveira & Sanches 2003).

The first National Serological Survey to determine a baseline status was carried out in 2001 in children aged one to five years. The seroprevalence rate measured in 14 among the 17 departments of the western region of Paraguay ranged from 0.1-1.4%, with highest seroprevalence rates of 1.2% and 1.4% being recorded in Paraguarí and Cordillera departments, respectively. A total of 10,406 children were screened in the 14 selected departments; 57 (0.5%) children among them were seropositive for a *T. cruzi* infection. Finally, for four among these 57 *T. cruzi*-confirmed seropositive children, the mothers were seronegative, and one child had received a prior blood transfusion. This information was documented to update the findings that had been gathered on the extent and the risk of the transmission of Chagas disease in Paraguay in 2001 (Peñaranda-Carrillo et al. 2002, OPS 2004).

The aim of the present study was to conduct the second National Serological Survey in children of the same age group and to determine the impact of entomological control and surveillance programs that ran between 2000 and 2007. The results may indirectly provide an assessment of the degree of risk for congenital transmission through the confirmation of seropositive cases and the possible identification of areas still at risk for vector transmission.

## MATERIALS AND METHODS

*Sampling framework* - Sampling procedures, conducted in 2008 in the rural areas of the 14 departments of the Oriental Region, followed those used for the 2007 population projection based on the data that had been gathered from the 2002 “National Census of Populations and Housing”. Using the defined criterion, districts in the departments without any rural areas were excluded from the sampling.

*Sample size* - The results of the first National Serological Survey carried out in 2001 in children aged one to five years were used to estimate the prevalence and confidence interval width in each department. The endemic and non-endemic status of each department was primarily based on data relative to the infestation rate of *T. infestans*, historical entomological data, and the baseline entomological surveys performed in the last five years. In addition, data related to seropositive pregnant women identified in prenatal screenings and data from blood bank registers were taken into account.

*Endemic departments and those with low endemicity* - The departments of Caaguazú, Itapúa, Caazapá, Central, Ñeembucú, Canindeyú, Amambay, Misiones, and Alto Paraná were considered areas with low endemicity. Sample size was calculated with a prevalence of 0.4% and a confidence interval (CI) width of 0.7%. The departments of Concepción, Cordillera, Paraguarí, San Pedro, and Guairá were considered historically endemic (infestation rates ranging from 0.4-2.8%, determined in the last five years prior to the present survey). Sample size was calculated with an estimated prevalence of 1.25% and a CI width of 1.5%.

*Sampling* - Each sample taken at random was processed in three stages. In the first stage, the localities in each department were selected at random with equal probability. In the second stage, households with children between one to five years of age were selected within each locality. In the third stage, a maximum of two children were selected from each household.

*Selection of localities* - To determine the number of localities to be selected in each department, the following considerations were taken into account: (1) the selection of the greatest number of localities possible (in order to achieve the widest distribution); (2) the sample size of children between one to five years of age to be selected in each department; (3) the proportion of children between one to five years of age in each department; (4) the variability of the number of households in each locality (according to the 2002 census); and (5) the 10% adjustment because of a possible non-response rate.

The population group between one to five years of age, according to the 2002 census, comprised 660,669 children in the 14 departments studied. The total number of existing localities was 4,245, of which 619 were selected for sampling (Table I).

**TABLE I:** Number of cases sampled in departments and seroprevalence of *Trypanosoma cruzi* in children aged one to five years living in rural areas of the Oriental Region of Paraguay

Nº	Department	Total population from 1-5 years old*	Sampling		

			Districts (nº)	Localities (nº)	Samples (nº)
1	Concepción	25,907	6	35	856
2	San Pedro	46,822	12	30	820
3	Cordillera	31,320	17	35	819
4	Guaira	22,162	14	43	813
5	Caaguazu	61,667	15	30	893
6	Caazapa	20,182	10	50	1,183
7	Itapua	64,437	16	30	904
8	Misiones	12,931	10	70	1,063
9	Paraguari	26,735	15	36	814
10	Alto Parana	90,375	14	32	907
11	Central	209,792	10	35	912
12	Ñeembucu	8,107	16	88	800
13	Amambay	15,531	3	75	1,157
14	Canindeyú	24,701	8	30	835
		660,669	166	619	12,776

*: projection of the population for the year 2007 based on the census of 2002.

*Selection of households* - The distribution of samples in each locality was carried out in proportion to the number of children between one to five years of age reported in the 2002 census, taking 10 households as the minimum number of households to be surveyed. When the number of children in 2002 equalled less than 10, we considered the total number.

*Selection of children* - From each household selected, we first chose one child aged one to five years, but only if he/she had been born and raised in that locality. When only one or two children from that age range were present in the household, one or both were utilised for the survey. If the number of children from that age range was greater than two, only two among them were randomly selected for the survey.

*Sample collection and serological studies* - Having obtained prior consent from parents or guardians of the children, we collected blood samples by digital puncture with a lancet onto filter paper and proceeded to draw up an epidemiological chart. The samples were stored in a refrigerator, and the cold chain was maintained when they were transferred. The detection of anti- *T. cruzi* immunoglobulin G (IgG) antibodies in the blood, collected on the filter paper, was carried out by enzyme-linked immunosorbent assay (ELISA) using the “IICS-Chagas” kit as a first step. This kit has been produced at the IICS since 1986. The “IICS-Chagas” kit utilises Y strain epimastigotes crude extract and goat anti-human IgG-peroxidase conjugate; it has a sensitivity (95% CI) of 97.02 (93.2-99.0) and a specificity (95% CI) of 99.24 (97.3-99.9) (Otani et al. 2009, do Brasil et al. 2016). The standardisation in the filter paper (Schleicher & Schuell, Germany) was performed in the IICS, and it has been used by our group since 1998 in children (Russomando et al. 2005). Briefly, one drop of blood is collected in a standardised circle, cut with a punch, and placed in 250 µL of the ELISA kit incubation buffer, overnight at 4ºC.

The maximum amount of time needed for processing, analysis, repetition, and confirmation was 30 days. We reconfirmed suspected positive cases with a second sample drawn by venipuncture that we tested by the conventional techniques of ELISA “IICS-Chagas” and indirect immunofluorescence test (IIF). The IIF reagents consisted of Y strain epimastigotes of *T. cruzi* and anti-human IgG-fluorescein conjugate. The cut off for IIF was a dilution of 1/20.

## RESULTS

*Samples collected on filter paper* - From January to April 2007, a total of 12,776 blood samples were collected from one to five-year-old children distributed in 619 localities in 166 districts of 14 departments of the Oriental Region of Paraguay. In this sampling, 52.2% of the children were female and 47.8% male, and the number sampled per age was very representative within the selected range (Table II).

**TABLE II:** Distribution by department, gender and age of 12,776 children aged one to five years tested for *Trypanosoma cruzi* infection in 2008

Nº	Department	Samples tested (nº)	Gender		Age (in years)				
	
			M	F	1	2	3	4	5
1	Concepción	856	465	391	128	122	133	203	270
2	San Pedro	820	440	380	153	194	201	261	11
3	Cordillera	819	405	414	112	137	231	215	124
4	Guaira	813	413	400	218	191	179	222	3
5	Caaguazu	893	451	442	144	205	227	297	20
6	Caazapa	1,183	659	524	209	242	287	323	122
7	Itapua	904	455	449	139	145	195	212	213
8	Misiones	1,063	541	522	143	155	205	250	310
9	Paraguari	814	435	379	143	151	159	212	149
10	Alto Parana	907	470	437	115	181	163	225	223
11	Central	912	495	417	115	179	204	301	113
12	Ñeembucu	800	430	370	107	141	173	234	145
13	Amambay	1,157	608	549	183	226	216	216	316
14	Canindeyú	835	418	417	139	175	206	202	113
		12,776	6,685	6,091	2,048	2,444	2,779	3,373	2,132

*Collection of venous blood and reconfirmation of positive cases* - A total of 41 positive and borderline *T. cruzi*-infected children were identified in the initial testing. Venous blood samples were subsequently drawn from both suspected children and their mothers.

The infection was confirmed in 30 (0.24% seropositive) among the 12,776 children tested. All seropositive children had *T. cruzi*-seropositive mothers*.* The entomological search for triatomines in the households with cases of a child testing positive, and none having received a prior blood transfusion, proved to be negative. Mothers received counselling after they were notified that both they and their children were infected.

*Seroprevalence rates in endemic and low endemic departments for children under five years following the serological survey (Table III)* - With a confidence level of 95%, endemic and low endemic departments showed average prevalence rates of 0.36% and 0.17%, respectively. The prevalence rates are significantly lower than the results obtained in the national survey from 2001 that reported average prevalence rates of 0.84% and 0.44%, respectively.

**TABLE III:** Number of cases sampled in departments and seroprevalence of *Trypanosoma cruzi* in children aged one to five years living in rural areas of the Oriental Region of Paraguay. Comparison of seroprevalence determined in 2001 and 2008 surveys

Departments	Infestation rate (*Triatoma infestans*) Years (1999-2005)	Blood bank year 2002	Samples and seroprevalence of *T. cruzi* in children aged from 1-5 years old (nº)					

			Survey in 2001			Survey in 2007		
	
			Samples (nº)	Seropositives (nº)	Sero-prevalence rate	Samples (nº)	Seropositives (nº)	Sero-prevalence rate
Concepción	1.2%	NA	452	2	0.44%	856	1	0.12%
Cordillera	1.3%	7%	433	6	1.38%	819	4	0.49%
Paraguari	2.8%	10%	436	5	1.15%	814	4	0.49%
San Pedro	0.4%	1.4%	484	3	0.61%	820	5	0.61%
Guaira	0.4%	2.7%	922	7	0.76%	813	1	0.12%
Caaguazu	0.2%	3%	934	6	0.64%	893	2	0.22%
Amambay	0.2%	NA	884	2	0.23%	1157	2	0.17%
Misiones	0.08%	0.8%	848	1	0.12%	1063	0	0.00%
Caazapa	0.05%	1%	932	1	0.11%	1183	1	0.09%
Central	< 0.05%	4.6%	852	3	0.35%	912	2	0.22%
Itapua	< 0.05%	1.4%	933	5	0.54%	904	2	0.22%
Ñeembucu	< 0.05%	3.5%	583	6	1%	800	2	0.25%
Canindeyu	< 0.05%	NA	811	6	0.74%	835	2	0.24%
Alto Parana	< 0.05%	2.5%	902	4	0.44%	907	2	0.22%
			10,406	57	0.55%	12,776	30	0.24%

NA: not available

## DISCUSSION

This present survey reports an estimated seroprevalence rate of 0.24% for a *T. cruzi* infection in children under five years old among the country’s rural population. The fact that only 30 (0.24%) cases among the 12,776 children tested were seropositive for a *T. cruzi* infection is a strong indication that the domiciliary transmission by *T. infestans* in the Oriental Region of Paraguay has been interrupted. This statement is supported by the following epidemiological data obtained in the present study: 100% of the children had never received a prior blood transfusion, 100% of the mothers of the infected children were also seropositive for *T. cruzi* (i.e., high risk for congenital transmission), and 100% of the infected children were born and raised in the same locality (under entomological surveillance since 2001). Additionally, no triatomines were detected in the dwellings at the moment of the survey.

We assume that *T. cruzi* was transmitted to these children vertically. The total number of infected children, aged one to five years living in these departments (Table II) was estimated at 1,691 cases with an annual incidence of congenital transmission of 338 cases per year. In the first National Serological Survey conducted in 2001 on the same age group, 57 (0.55%) among 10,406 children were seropositive and four had been infected by vector transmission. It was calculated that the number of children, in this age group, infected through vertical transmission was almost twice the number of cases detected seven years earlier, i.e., 3,411 infected children in 2001. With an estimated annual incidence of 682 cases per year, it is important to point out that in the first survey the seroprevalence ranged from 0.1-1.4%.

If we compare the results obtained in both surveys, we can observe in the more recent survey a near 50% reduction in: (i) the total number of infected children; (ii) the number of estimated cases of congenital transmission; and (iii) the annual incidence of vertical transmission. Given these results, we can determine the impact of vector control in the transmission of *T. cruzi*, following uninterrupted vector control measures applied since 1999 in contiguous *T. infestans*-endemic areas of Paraguay. Next, we can estimate the degree of risk of congenital transmission. Based on the technical report on entomological surveillance in the national territory that showed the presence of very few residual foci of *T. infestans* in historically endemic localities (OPS 2006) and the results obtained in this study, in June 2008, Paraguay received the “international certification of the interruption of domiciliary transmission of Chagas disease by *T. infestans* in the Oriental Region” (MSPBS 2008, OPS 2008).
